# Histamine causes endothelial barrier disruption via Ca^2+^-mediated RhoA activation and tension at adherens junctions

**DOI:** 10.1038/s41598-018-31408-3

**Published:** 2018-09-05

**Authors:** Daniela Kugelmann, Lukas Thomas Rotkopf, Mariya Yosifova Radeva, Alexander Garcia-Ponce, Elias Walter, Jens Waschke

**Affiliations:** Institute of Anatomy and Cell Biology Department I, Ludwig-Maximilians-Universität München, Munich, Germany

## Abstract

During inflammation, the disruption of the endothelial barrier leads to increased microvascular permeability. Whether tension along cell junctions contributes to histamine-induced endothelial barrier disruption remains unknown. Rapid Ca^2+^ influx induced by both histamine and thrombin was accompanied by endothelial barrier breakdown revealed as drop of transendothelial electric resistance in primary human microvascular endothelial cells. Interestingly, GLISA measurements revealed activation of RhoA but not inactivation of Rac1 at the time-point of barrier breakdown. FRET measurements showed activation of RhoA at intercellular junctions after both thrombin and histamine exposure. Breakdown coincided with increased stress fiber formation but not with translocation of vinculin, which was located along junctions in the resting state similar to postcapillary venules *ex vivo*. Moreover, increased tension at AJs was indicated by immunostaining with a conformation-sensitive antibody targeting the α18-subunit of α-catenin. Ca^2+^ chelation by BAPTA-AM and ROCK1 inhibition by Y27632 abolished both increase of tension along AJs as well as barrier dysfunction. Moreover, BAPTA-AM decreased RhoA activation following histamine stimulation, indicating a key role of Ca^2+^ signaling in barrier breakdown. Taken together, in response to histamine, Ca^2+^ via RhoA/ROCK activation along endothelial adherens junctions (AJs) appears to be critical for barrier disruption and presumably correlated with enhanced tension. However, vinculin appears not to be critical in this process.

## Introduction

The endothelium provides a selective barrier between the blood and the surrounding interstitial tissue providing nutrients to the tissues, regulating vascular homeostasis and the transmigration of leukocytes, preserving tissues through oncotic pressure control and participating in tumor neoangiogenesis and inflammatory reactions^1–4^. Endothelial cells are highly dynamic and are constantly subject to changes due to mechanical forces, and endothelial cell junctions together with the glycocalyx form the endothelial barrier^5^. Within the intercellular contacts, tight junctions (TJs) directly limit paracellular permeability whereas adherens junctions (AJs) mechanically couple neighboring cells^6^. Both junction types are functionally linked with the cortical actin cytoskeleton through several adaptor molecules. Therefore, intracellular signaling affecting actin dynamics is essential in regulating endothelial barrier function^7–10^.

At the molecular level, AJs in endothelial cells are composed of vascular endothelial (VE-) cadherins, which allow for intercellular linkage with their extracellular domains in a Ca^2+^-dependent homophilic manner^11^. VE-cadherin is also crucial for endothelial barrier maintenance and recovery. The cytoplasmic domain of the VE-cadherin tail has binding sites for p120 catenin, which has been indicated to control VE-cadherin turnover, lateral clustering and junction integrity^8^, as well as ß-catenin and y-catenin, which, subsequently bind to α-catenin, sequentially, linking the whole complex directly and/or indirectly via adaptor molecules to the actin cytoskeleton^12–15^. Cadherin complexes have been shown to not only transmit force but also act as active mechanosenors^16^. In this context, the adaptor protein vinculin, which anchors the cadherin complex via α-catenin to the actin cytoskeleton, is involved^17–20^. Vinculin interaction with AJ proteins protects VE-cadherin junctions from opening during their force-dependent remodeling^16,21^. One of the most potent signaling pathways for stabilization of the barrier properties is regulation of actin dynamics through cyclic adenosine monophosphate (cAMP)^7^. Furthermore, small GTPase family members, especially Rac1, Cdc24 and RhoA are crucial for regulation of the endothelial barrier^3,5,7,22^. Therefore, a fine balance between the activities of RhoA and Rac1 GTPases is required to maintain AJs^23^. Interestingly, we demonstrated in a former study that cAMP stabilizes the endothelial barrier via Rac1. Rac1 activation stabilizes the endothelial barrier against vasoactive stimuli *in vitro* and *in vivo*^24,25^, whereas RhoA has a diverse function in endothelial barrier regulation^5,26^.

RhoA effectively modulates the organization and dynamics of the actin cytoskeleton^11^. Furthermore, RhoA was found to induce the disassembly of endothelial AJs in response to inflammatory conditions^23^. Vasoactive mediators such as lipopolysaccharide (LPS) or tumor necrosis factor alpha (TNF-α) inhibit cAMP/Rac1 signaling and cause strong activation of RhoA^5^. RhoA activation leads to breakdown of the barrier, which is accompanied by the formation of gaps and stress fibers. This breakdown is caused by opening of interendothelial junctions in postcapillary venules which results in severe subcutaneous and whole-body cavity edema^3^. Although considerable research interest in the mechanisms underlying barrier disruption is evident, the significance of tension generation during acute barrier breakdown is still unclear. In this study, we used the inflammatory mediator histamine, which has been shown to regulate endothelial properties through activation of the type I histamine receptor and Rho A signaling^27,28^, to study the mechanisms involved in anaphylaxis in primary human microvascular endothelial cells *in vitro*, and microvascular rat mesentery vessels *in vivo*. Here, we propose that histamine-mediated disruption of endothelial barrier function is Ca^2+^- and Rho kinase-dependent and correlates with enhanced tension at AJs.

## Results

### Histamine and thrombin induce AJ disruption and barrier dysfunction

**Figure 1:**
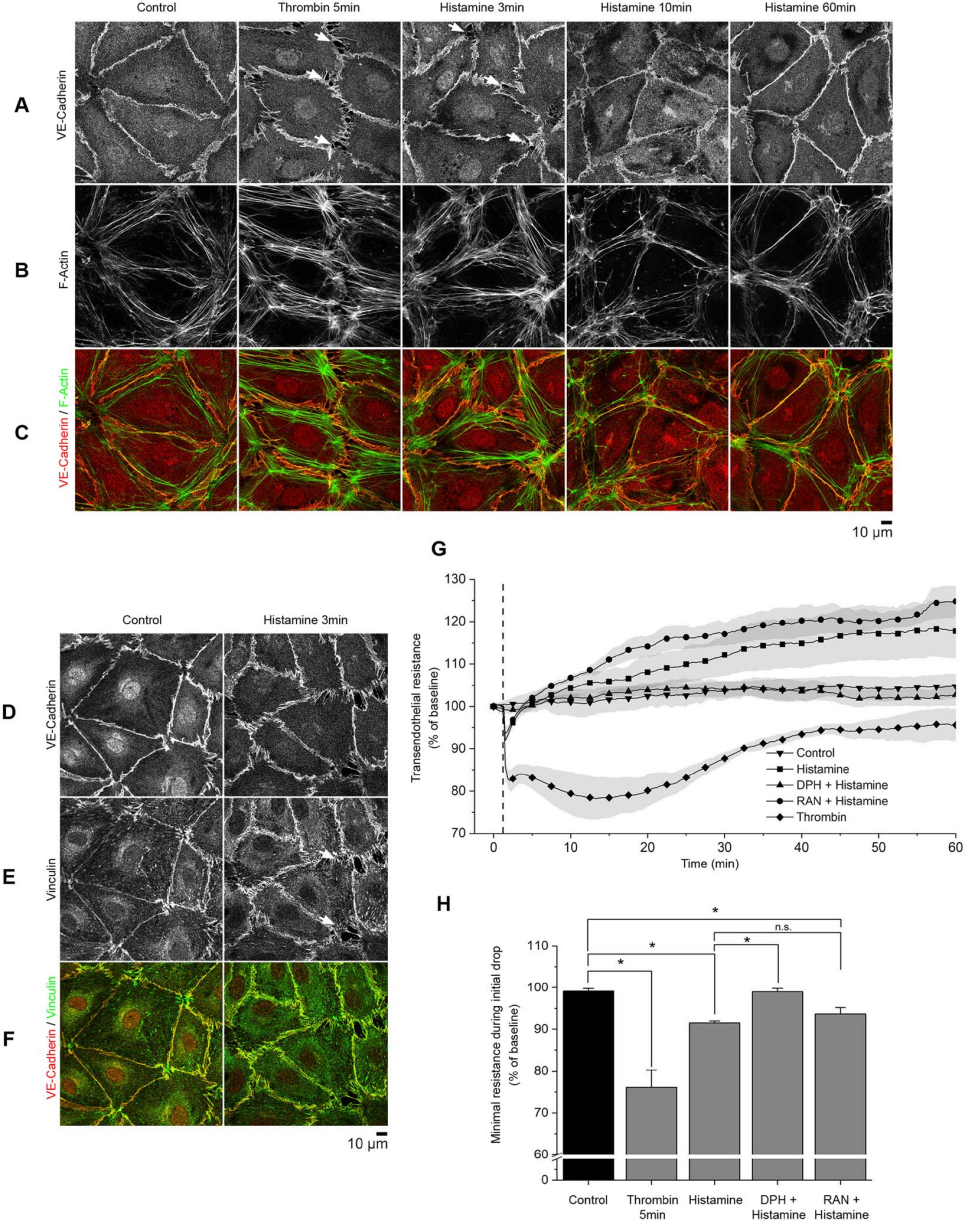
Changes in endothelial morphology and barrier integrity induced by inflammatory mediators. Confluent HDMEC monolayers were incubated with either histamine or thrombin and subsequently fixed with PFA (representative images of n = 4 independent experiments). (**A**–**C**) Immunostaining against VE-cadherin and F-actin. Histamine and thrombin induced gap formation as indicated by VE-cadherin staining (arrows) and increased stress fiber formation after 3 and 5 min, respectively. At 60 min, stress fiber formation was largely abolished. (**D**–**F**) Co-staining for VE-cadherin and vinculin under control conditions and after incubation with histamine for 3 min. Vinculin was visible at the membrane under both conditions, however gap formation was present only upon histamine treatment (arrow). (**G**) TER measurements in confluent HDMECs after histamine or thrombin stimulation with or without pre-incubation with diphenhydramine (DPH) or ranitidine (RAN) for 60 min (n = 4 independent experiments, *p < 0.05, dotted line indicates application of mediators). (**H**) Quantification of minimal resistance during the initial drop showed decreases in basal resistance to 93.5% ± 0.8% (histamine) and 78.6% ± 3.7% (thrombin). Pre-incubation with DPH reduced the drop significantly to 98.6% ± 0.9% whereas pre-incubation with RAN had no significant effect.

Barrier function in endothelial cells is controlled by AJs and TJs, which are disrupted during pathological states^29^. To elucidate the time course under which endothelial monolayers react to the short-term vasoactive mediators, histamine and thrombin, immunofluorescence analysis of vascular endothelial cadherin (VE-cadherin), filamentous actin (F-actin), and vinculin was performed at several time points. After the addition of either histamine for 3, 10 and 60 min or thrombin for 5 min, confluent HDMEC monolayers were fixed and stained for VE-cadherin (Fig. 1A) and labeled for F-actin using Alexa 488 phalloidin (Fig. 1B). Consistent with reports in the literature^27^, short-term incubation with thrombin and histamine led to intercellular gaps- and increased stress fiber formation. Three minutes after histamine addition, intercellular gaps formed (Fig. 1A, arrows) and increased stress fiber formation was detected (Fig. 1B). Thrombin led to similar but stronger effects after 5 min of incubation. Ten minutes after histamine incubation, the intercellular gaps had largely closed. After 60 min of incubation, stress fiber formation was barely visible, and the intercellular gaps were no longer detectable (Fig. 1A–C). In contrast to control conditions, during the initial response, the linear formation of VE-cadherin at the intercellular membrane was disrupted and VE-cadherin distribution appeared a ragged (Fig. 1A). Furthermore, staining of vinculin and VE-cadherin in HDMECs under control conditions or after 3 min of histamine incubation (Fig. 1D–F) showed that vinculin staining was detectable on the cell membrane under both control and inflammatory conditions and that vinculin largely co-localized with VE-cadherin (Supplementary Fig. S1A).

In parallel, endothelial breakdown was detected by transendothelial electric resistance (TER) measurements. For that purpose, HDMECs were grown to confluence and then incubated with histamine or thrombin. Histamine induced subtle and transient barrier dysfunction and basal resistance was significantly reduced to 93.5% ± 0.8% after 0.5 min. Recovery to baseline was evident after 3.5 min. Then, TER continued to rise above control values, which were stable for the 60 min incubation period. In contrast, thrombin induced a stronger drop in TER (78.6% ± 3.7%), which recovered more slowly. In addition, specific inhibitors of histamine receptor type I, diphenhydramine (DPH) and histamine receptor type II, ranitidine (RAN) were preincubated with HDMECs for 15 min before the addition of histamine. As expected, diphenhydramine blocked the initial resistance drop whereas ranitidine had no significant effect (Fig. 1G,H).

### Increased α18-catenin staining indicates higher tension along AJs during the initial histamine response

**Figure 2:**
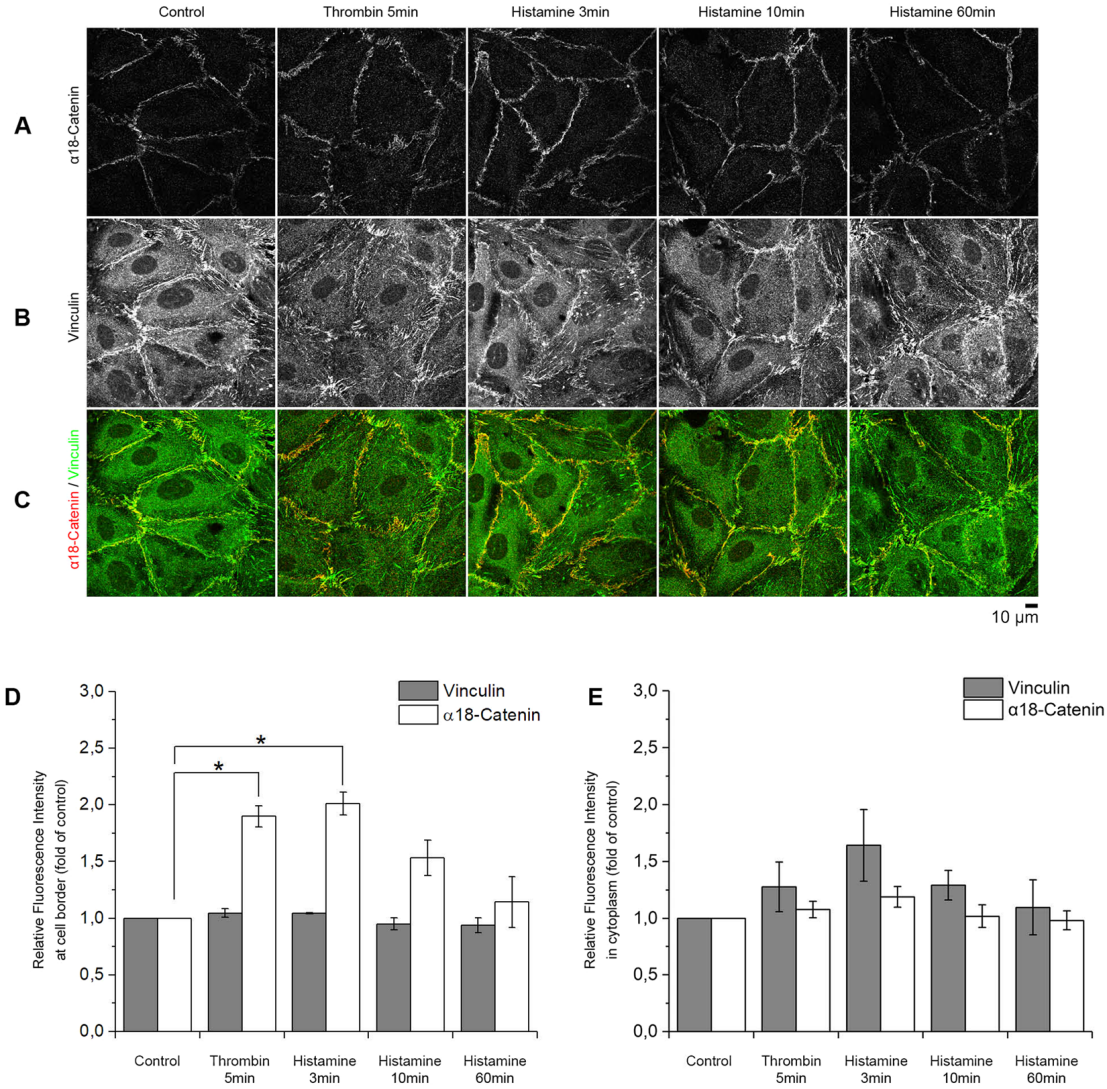
α18-catenin staining along AJs. (**A**–**C**) Staining with antibodies against α18-catenin and vinculin after the addition of histamine or thrombin at different time-points. (**D**) Automated quantification of the fluorescence intensity at the cell border after staining with α18-catenin or vinculin using CellProfiler. Intensity of α18-catenin staining increased significantly 3 and 5 min after addition of histamine or thrombin, respectively (n = 4 independent experiments, *p < 0.05). At these time points intensity of vinculin staining did not change significantly at the membrane. (n = 4 independent experiments, *p < 0.05) The relative fluorescent intensity of vinculin in the cytoplasm was slightly increased upon thrombin and histamine treatment while under the same conditions α18-catenin was unaltered. (n = 4 independent experiments, *p < 0.05).

A conformation-sensitive antibody targeting the α18-subunit of α-catenin (α18-catenin) was used. It has been described that antibody binding correlates with tension at AJ because the epitope it binds to, is exposed during tension^30^. Morphologically, stronger but discontinuous staining of α18-catenin primarily along cell borders was detectable after 3 min of histamine incubation compared to control (Fig. 2A). Staining of vinculin at the cell border was observed under control conditions and colocalization with α18-catenin did not differ significantly after addition of histamine (Fig. 2B, Supplementary Fig. S1B). Quantifications of the relative fluorescence intensity revealed that the mean changes in α18-catenin-staining after histamine addition were 201.1% ± 10.0%, 152.9% ± 15.6% and 114.3% ± 22.4% of control, after 3, 10 and 60 min, respectively, whereas thrombin increased α18-catenin-staining by 189.7% ± 9.2% at the border (Fig. 2D) but not in the cytoplasm (Fig. 2E). In contrast, the fluorescence intensity of vinculin did not significantly change at the cell borders or cytoplasm (Fig. 2D,E). In addition, histamine transiently induced a dotted staining pattern resembling focal adhesions which was reflected in a not significant increase of vinculin staining intensity in the cytoplasm and which was also detectable following treatment with thrombin.

### Activation of Rho GTPases and Ca^2+^ influx under inflammatory conditions

**Figure 3:**
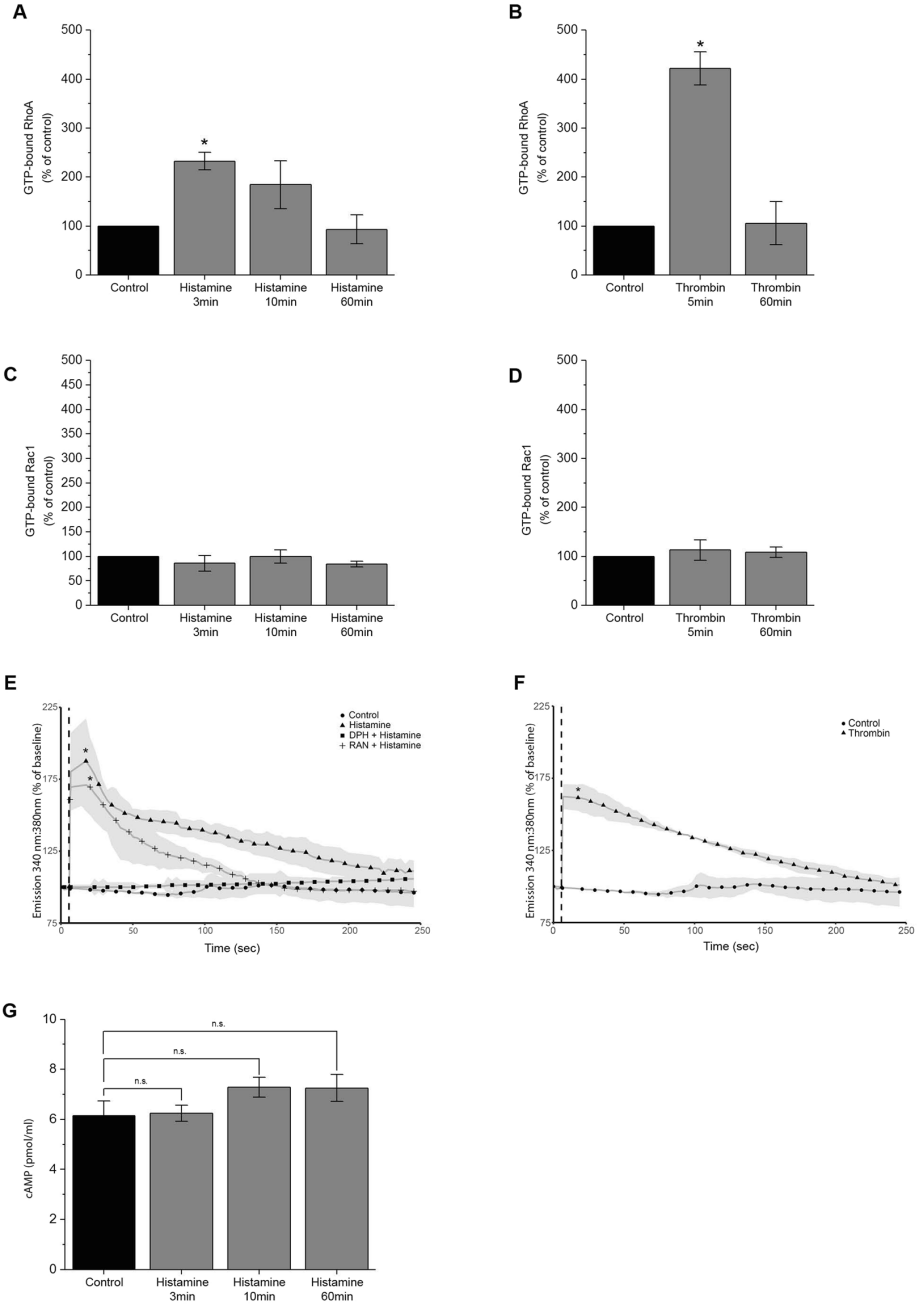
Signaling pathways activated during histamine-mediated barrier disruption. (**A**,**B**) RhoGTPase ELISA (GLISA) measurements of HDMECs after incubation with histamine or thrombin at different time points. In comparison to the control, RhoA activity increased significantly 3 and 5 min after addition of histamine or thrombin, respectively (n = 4 independent experiments, *p < 0.05). (**C**,**D**) No changes of Rac1 activity, measured by GLISA, were observed upon different treatments (n = 3 independent experiments, *p < 0.05). (**E**) Fluorescence intensity measurements of FURA-2-AM revealed a significant increase in intracellular Ca^2+^concentration following histamine stimulation. Pre-incubation with DPH but not RAN completely abolished Ca^2+^ influx (n = 5 independent experiments, *p < 0.05, dotted line indicates histamine application). (**F**) FURA-2-AM measurements after thrombin stimulation showed a similar burst in intracellular Ca^2+^ levels (n = 4 independent experiments, *p < 0.05, dotted line indicates addition of thrombin). (**G**) Measurement of intracellular cAMP concentration after histamine treatment revealed no significant change of cAMP levels (n = 4,*p < 0.05).

The ability of endothelial cells to contract involves myosin light chain phosphorylation via myosin light chain kinase (MLCK) and Rho family proteins^31^. Of these, RhoA and Rac1 have been reported to play major roles in the short-term inflammation response^26,27,32^. Intracellular GTP-bound Rac1 or RhoA levels were measured by GLISA after the addition of either thrombin or histamine following a standard protocol. GLISA measurements revealed significant activation of RhoA to 232.4% ± 17.9% of control after incubation with histamine for 3 min (Fig. 3A) and 421.8% ± 33.4% after 5 min of thrombin treatment (Fig. 3B). There were no significant changes after either incubation of histamine for 10 min or 60 min as well as thrombin for 60 min (Fig. 3A,B). Under the same conditions, no significant changes in Rac1 activity were detected (Fig. 3C,D). As cAMP levels allegedly contribute to barrier breakdown and recovery^20,33^, intracellular cAMP levels were measured by ELISA. However, no significant changes in cAMP concentrations were detected during the course of barrier breakdown and recovery (Fig. 3G).

After activation of the histamine type 1 receptor, downstream activation of PLC-ß leads to increased intracellular Ca^2+^ concentrations, subsequently activating a cascade of signaling molecules^23,34,35^. Ratiometric FURA measurements^36^ were used to determine the Ca^2+^-concentration in HDMEC monolayers after histamine or thrombin stimulation. Both mediators lead to a strong Ca^2+^ influx that correlated with force production. Preincubation with DPH abrogated this influx, whereas the histamine type 2 receptor inhibitor RAN did not (Fig. 3E,F).

### Histamine and thrombin cause activation of RhoA along cell junctions

**Figure 4:**
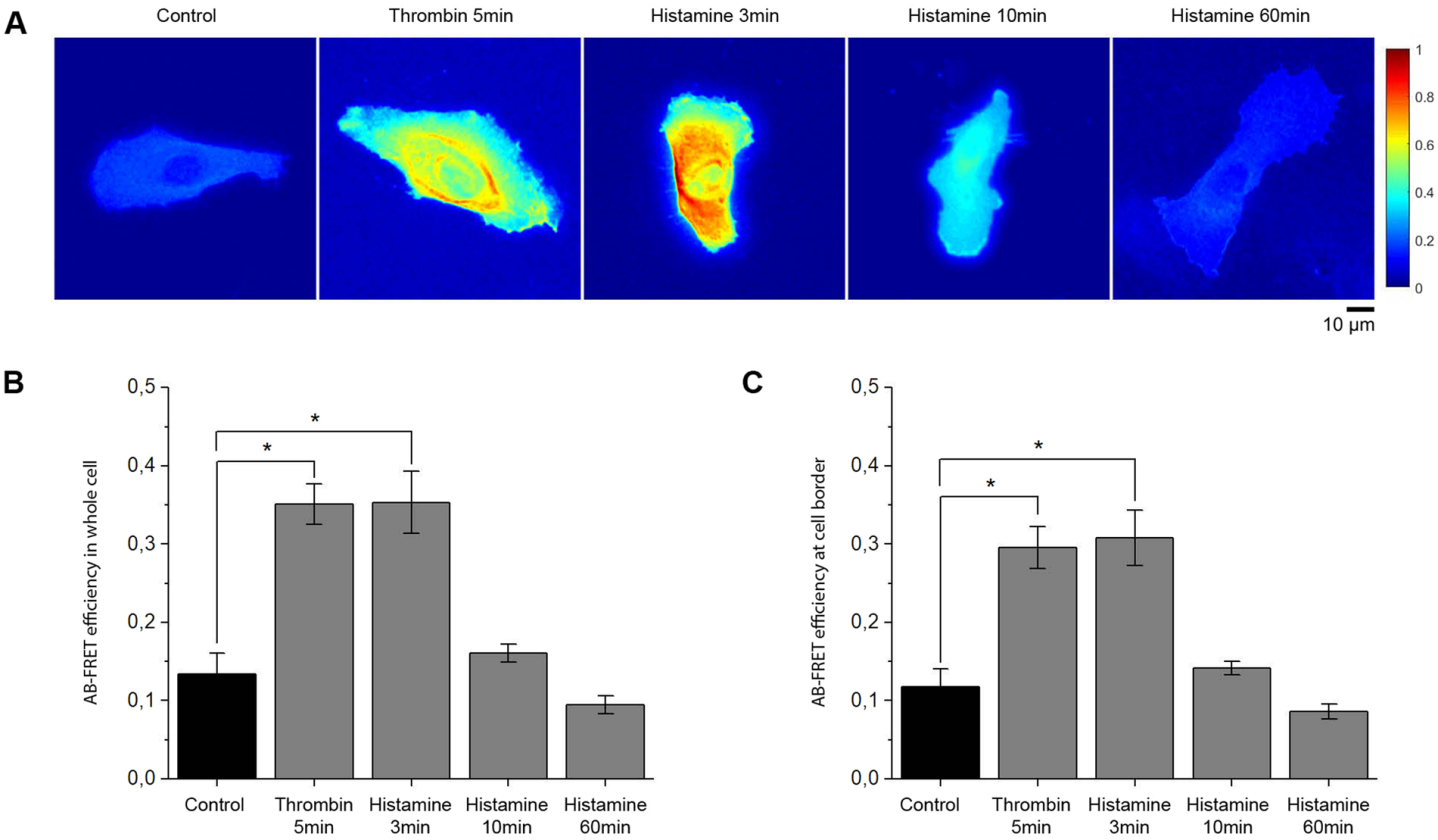
Histamine and thrombin induce activation of RhoA at cell borders. Acceptor-photobleaching FRET efficiency in HDMECs transfected with Raichu-RhoA probes and treated with either histamine or thrombin. (**A**) Pseudocolor representation of the absolute FRET efficiency showed localization of active RhoA in the whole cell (representative images of n = 5 independent experiments). (**B**,**C**) The mean absolute FRET efficiency quantified over the whole cell and cell borders using CellProfiler (n = 5 independent experiments, *p < 0.05). Incubation with thrombin (5 min) and histamine (3 min) significantly increased RhoA activity in the whole cell as well as at the cell border. (n = 5 independent experiments, *p < 0.05).

To further investigate the signaling patterns involved in histamine-induced inflammation, we used fluorescence resonance energy probes to elucidate the subcellular localization of GTP-bound RhoA^32^. Primary microvascular endothelial cells (HDMEC) were transfected with Raichu-RhoA plasmid and allowed 24 h of rest for stable expression of the Förster Resonance Energy Transfer (FRET) probes. Under basal conditions, the probe localized primarily to the membrane, but was also detectable throughout the cytosol (Fig. 4A). Consistent with the GLISA experiments, in the whole cell histamine and thrombin both increased the absolute FRET efficiency to 263.8% ± 29.5% and 262.0% ± 19.3% of control after 3 and 5 min, respectively (Fig. 4B). Furthermore, RhoA activity at the membrane was measured by averaging the FRET efficiency over the outermost ten pixels of the cell (Fig. 4C). We detected activation to 263.1% ± 30.0% and 252.2% ± 23.0% of control at the cell border after addition of histamine and thrombin, respectively. At the 10 and 60 min time points of histamine treatment, FRET efficiency no longer differed significantly from control.

### Inhibition of ROCK and Ca^2+^ -signaling reduces histamine-induced barrier dysfunction and α18-catenin staining

**Figure 5:**
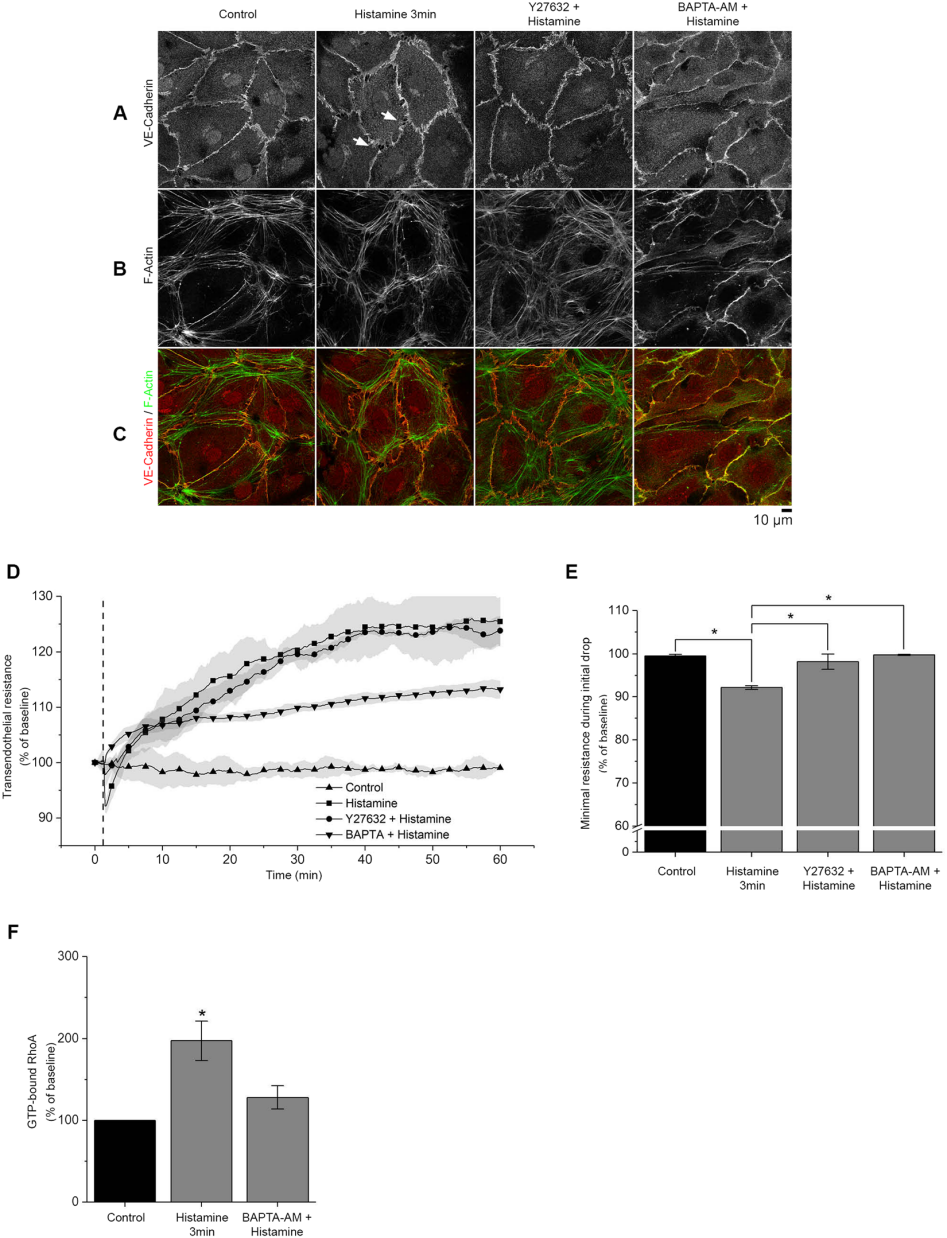
Inhibition of ROCK and Ca^2+^ signaling reduces histamine-induced barrier dysfunction. (**A**–**C**) Staining of HDMEC monolayers against VE-cadherin and F-actin after stimulation with histamine alone or after pre-incubation with either Y27632 (30 min) or BAPTA-AM (2 h). The gaps (arrows) induced by histamine (3 min) were largely abolished by application of either Y27632 or BAPTA-AM (n = 4 independent experiments). (**D**,**E**) TER measurements showed that the initial drop in resistance, induced by histamine, was significantly abolished by pre-incubation with both mediators (n = 3 independent experiments, *p < 0.05). (**F**) RhoA GLISA measurements in HDMECs revealed that BAPTA-AM reduces RhoA activation after histamine stimulation. (n = 3 independent experiments, *p < 0.05 vs control).

Downstream of histamine receptor activation, intracellular Ca^2+^ -signaling and Rho Kinase (ROCK) activation contribute to endothelial barrier dysfunction^27,28^. Y27632, a specific inhibitor of ROCK I and ROCK II and BAPTA-AM, an intracellular Ca^2+^-chelator, were used to suppress these pathways. Primary microvascular endothelial cells were preincubated with Y27632 or BAPTA-AM before histamine or thrombin addition and stained for VE-cadherin and F-actin (Fig. 5A–C, Supplementary Fig. S2A–C). Both Ca^2+^-chelation and ROCK inhibition prevented AJ disruption. Interestingly, BAPTA-AM effectively abrogated stress fiber formation whereas Y27632 did not, suggesting that Ca^2+^-dependent and Rho-independent pathways may also be involved. The results were confirmed by TER measurements (Fig. 5D). The drop in TER after the addition of histamine was inhibited by incubation with both BAPTA-AM (maximum drop 99.4% ± 0.3% of baseline) and Y27632 (maximum drop 98.1% ± 1. 8%) (Fig. 5E). However, GLISA experiments showed that preincubation with BAPTA-AM significantly reduced RhoA activation in response to histamine to 127.9% ± 14.4% of control compared to 197.2% ± 24.06% after incubation with histamine alone for 3 min, suggesting that Ca^2+^ -signaling is required for full activation of RhoA (Fig. 5F).

**Figure 6:**
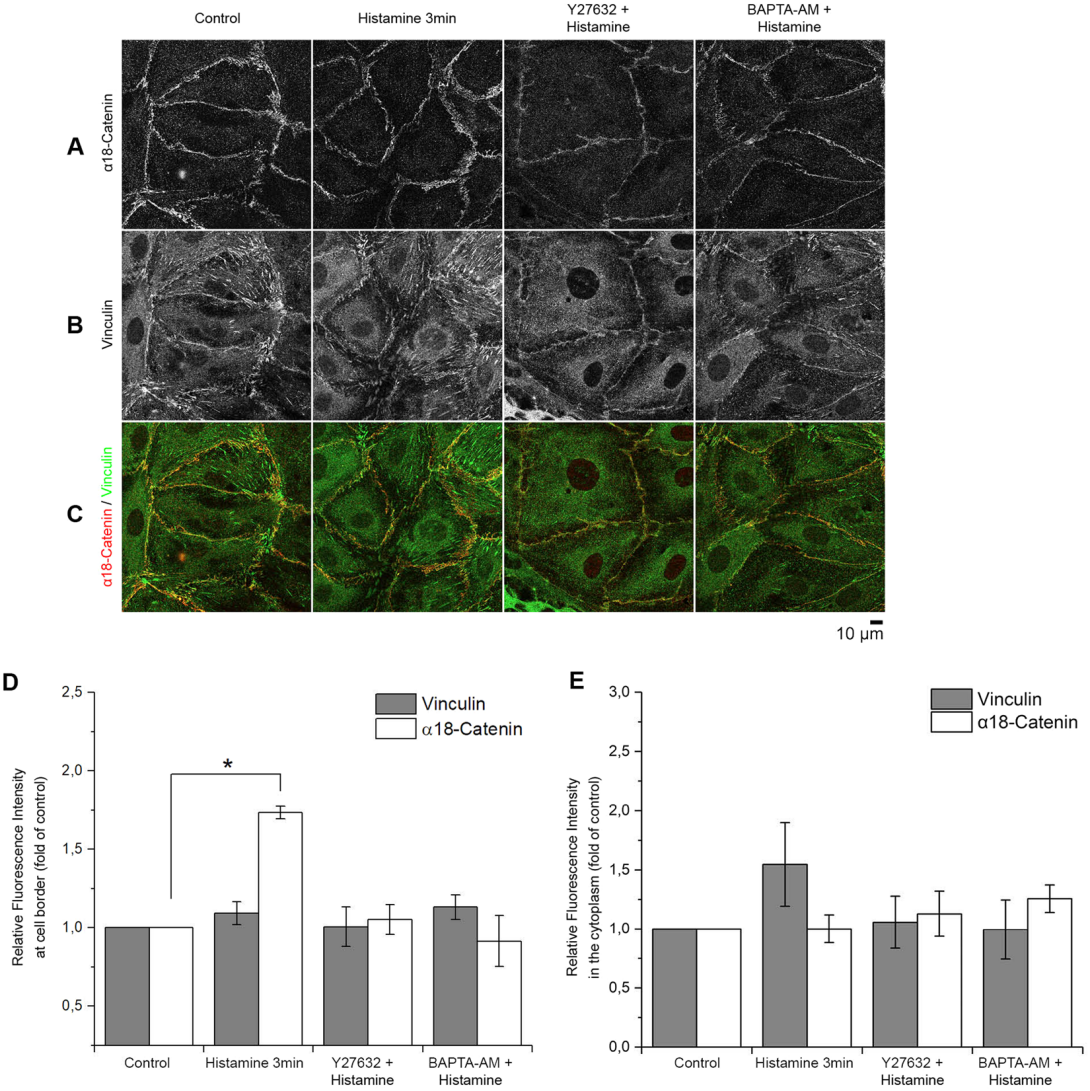
Increased α18-catenin staining along AJ is dependent on ROCK and intercellular Ca^2+^ signaling. (**A**–**D**) In HDMECs co-staining against α18-catenin and vinculin revealed a marked increase in α18-catenin staining intensity at cell borders following histamine incubation (3 min). However, this was not the case after pre-incubation with Y27632 or BAPTA-AM (n = 3 independent experiments). (**D**,**E**) Bar graph showing automated quantification of vinculin and α18-catenin fluorescence intensity at cell border and in the cytoplasm. Vinculin staining showed no significant change in localization or intensity after pre-incubation with Y27632 or BAPTA-AM. (n = 3 independent experiments, *p < 0.05).

Next, we studied the effect of ROCK inhibition and Ca^2+^-signaling on α18-catenin staining intensity along cell borders (Fig. 6A). The mean relative intensity in α18-catenin staining at cell borders after histamine incubation (161.2% ± 18.5%) was reduced by preincubation with Y27632 (106.2% ± 18.2%) and BAPTA-AM (99.6% ± 19.7%) (Fig. 6D). Notably, the intensity of vinculin staining at junctions as well as colocalization with α18-catenin did not change significantly (Fig. 6B,D,E, Supplementary Fig. S1C). In addition, similar to Fig. 2 histamine induced a dotted staining pattern of vinculin resembling focal adhesions which was reflected in an inconsistent but not significant increase of vinculin staining intensity in the cytoplasm and also was abrogated by and BAPTA-AM.

### Histamine disrupts actin and vinculin localization along endothelial membranes *in vivo*

**Figure 7:**
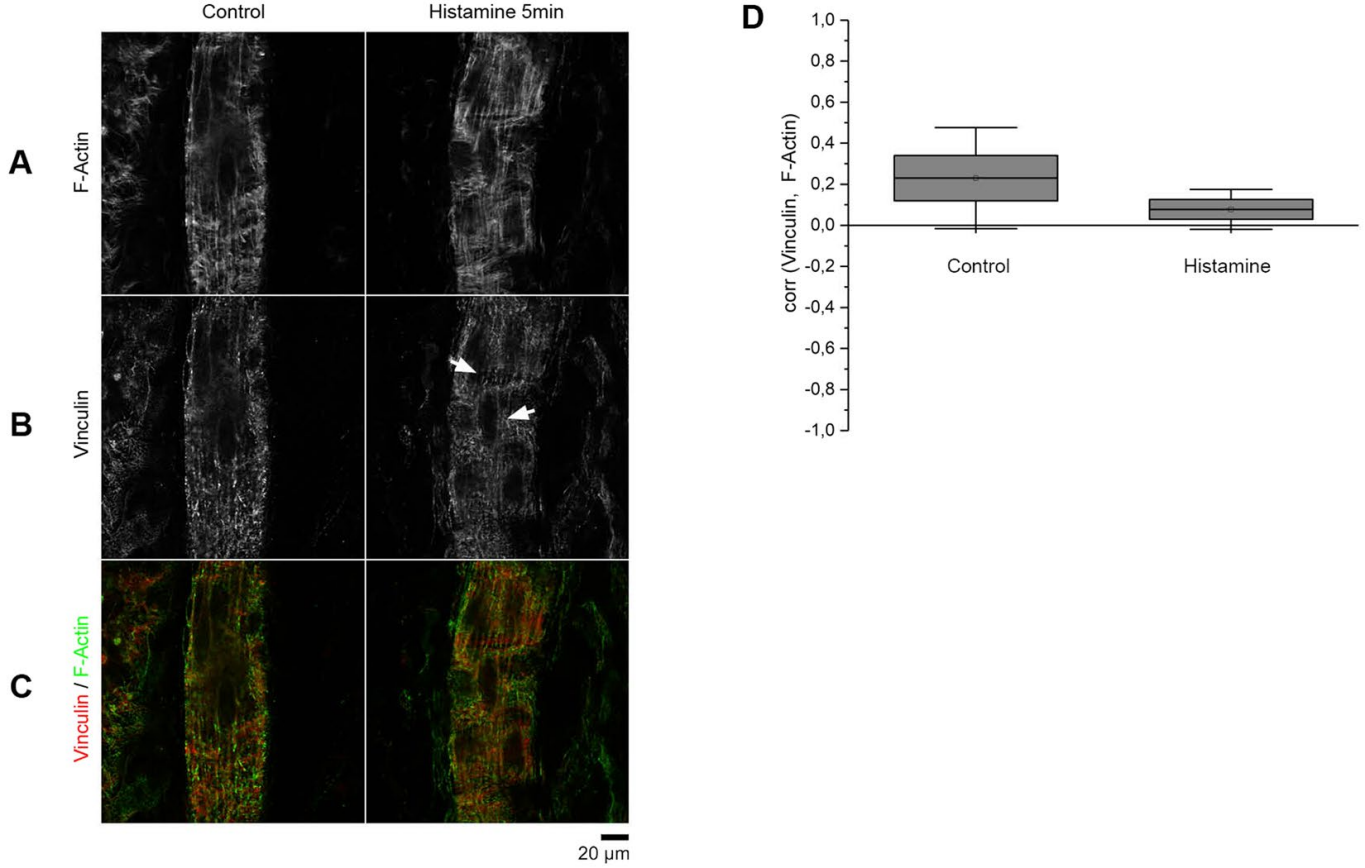
*Ex-vivo* immunostaining of single-perfused rat postcapillary venules demonstrate disruption of vinculin staining along the membrane after histamine stimulation. (**A**) In postcapillary venules actin forms longitudinal fiber bundles. (**B**) Endothelial cells display vinculin staining under resting conditions while the staining along cell borders is disturbed after stimulation with histamine (arrow). (**C**) Overlay of F-actin and vinculin staining (n = 4 independent experiments). (**D**) Co-localization between vinculin and actin, determined by Pearsons correlation coefficient, appears to be slightly reduced due to histamine treatment (n = 4 independent experiments).

To test whether the aforementioned effects of histamine also occur *in vivo*, the experimental conditions were replicated in a rat model. We used the modified Landis technique^1^, in which a single postcapillary venule of rat mesentery was cannulated with a glass capillary and continuously perfused with ringer solution containing BSA to dilute erythrocytes to visualize flow. After 5 min of perfusion with either control or histamine-containing solution the rat was euthanized, and the mesentery was immediately fixed with formaldehyde. The injected and marked venules were immunostained for vinculin together with F-actin labeling. Unfortunately, α18-catenin staining was not feasible since the antibodies origin is rat. Of note, parallel experiments using thrombin were not possible since it is known that intact mesenteric venules do not respond to thrombin^37,38^. Under control conditions, we observed colocalization of actin and vinculin along the endothelial cell membranes (Fig. 7). Endothelial cell borders were smooth and parallel to the flow direction. After perfusion with histamine for 5 min, vinculin staining alongside the endothelial cell borders changed to a more ragged appearance (Fig. 7A–C). These results indicate that similar to primary cultured endothelium, vinculin was present at cell borders under resting conditions. Upon histamine treatment, we observed a trend towards lower colocalization of vinculin and filamentous actin as measured by Pearsons’ correlation coefficient, which however was not significant (Fig. 7D).

## Discussion

### Histamine induces a transient decrease in barrier function accompanied by increased α18-catenin staining along AJs

The signaling mechanisms underlying acute endothelial barrier breakdown under inflammatory conditions are not entirely clear. Histamine and thrombin induce acute break-down of the endothelial barrier. However, thrombin is not a typical inflammatory mediator since it regulates vascular permeability during the coagulation phase after injury^39^ and in many studies macrovascular endothelial cells were used, which are good models for investigating adaptations to sheer stress but not the inflammatory response, which occurs exclusively in postcapillary venules^3^. Therefore, re-evaluation of all findings using microvascular endothelium is necessary. Histamine impaired the integrity of the endothelial barrier *in vitro* and *in vivo*^23,40–42^. The mechanism by which histamine leads to the disruption of the endothelial barrier is not fully understood, however, H1R Gαq-coupled receptors^27,42,43^ and PCK-, RhoA/ROCK- and NO-signaling seem to be important. In addition, histamine has been proposed to disrupt AJs, leading to the rapid formation of a new junction type referred to as focal adherens junction (FAJ), which is characterized by recruitment of vinculin^16^.

**Figure 8:**
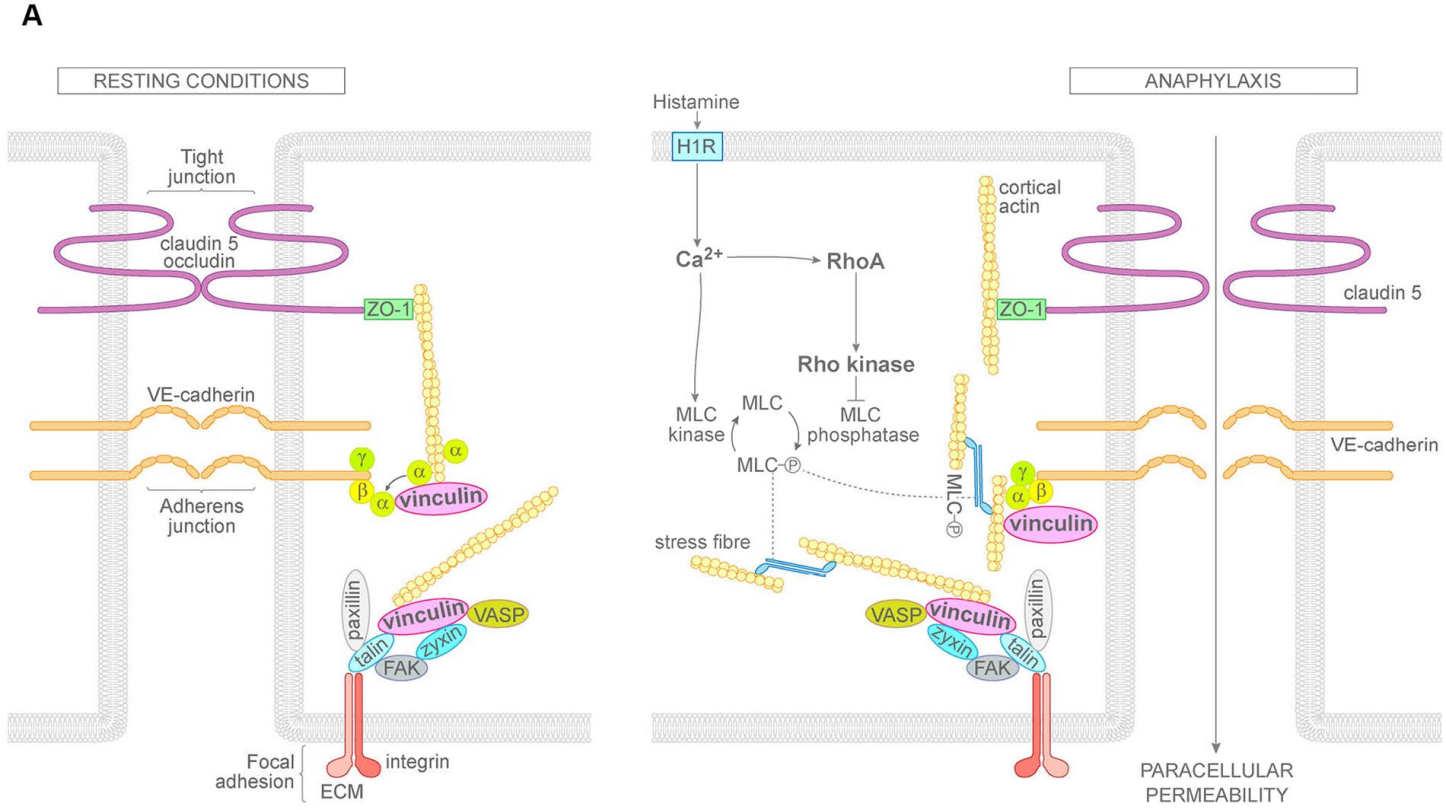
Mechanisms of histamine-induced endothelial barrier disruption. Under resting conditions, vinculin is localized mainly along endothelial cell borders and at least in part co-localizes with VE-cadherin and α-catenin at AJs. In anaphylaxis, histamine induces a rapid increase of cytoplasmic Ca^2+^ leading to activation of RhoA. Both events induce stress fiber formation at focal adhesions. In addition, RhoA via Rho kinase may enhance tension on α-catenin at endothelial AJs which causes AJ reorganization. H1R = Histamine receptor type I; Ca^2+^ = calcium; MLC = myosin light chain; FAK = focal adhesion kinase; VASP = vasodilator-stimulated phosphoprotein; ZO-1 = zonula occludens protein1; α, β, γ = α-, β-, γ-catenin; ECM = extracellular matrix.

In this study, we used histamine to study barrier disruption and the corresponding signaling pathways involved in anaphylaxis in primary human microvascular endothelial cells *in vitro* and postcapillary venules of rat mesentery *in vivo*. Thrombin was used in comparison *in vitro* only because intact mesenteric venules do not respond to thrombin^37,38^. Consistent with the literature^42^, our data showed that histamine and thrombin disrupted the endothelial barrier transiently and caused reduced TER and intercellular gap formation. At the same time, both thrombin and histamine enhanced staining with the conformation-sensitive antibody targeting the alpha 18-subunit of catenin which has been proposed to correlate with increased tension at AJs (Fig. 8). Increased tension may result from RhoA-dependent up-regulation of actomyosin contraction^27^ which may trigger relocalization of vinculin to AJs from FAJs^16^. Therefore, we correlated the different events during endothelial barrier breakdown with immunostaining for vinculin, which is known to be a mechanotransducer with the ability to stabilize adhesion under force^16,44–46^. Interestingly, we observed that vinculin was localized at cell junctions under resting conditions both in HDMECs *in vitro* and endothelial cells of postcapillary venules *in vivo*. Moreover, after incubation with either histamine or thrombin, vinculin distribution paralleled VE-cadherin and F-actin localization. AJs similar to vinculin were reorganized from continuous linear distribution towards a fragmented staining pattern with perpendicular arrays which were previously described as FAJ. However, the staining intensity for vinculin remained unaffected. These data indicate that in microvascular endothelium, vinculin is a normal constituent of AJs under resting conditions (Fig. 8) and that the term FAJ may not be adequate in this cell type. The discrepancy to the previous study^16^ may be explained at least in part by the fact that most experiments in the previous study were shown for HUVECs which is a macrovascular endothelial cell type from fetal tissue and therefore is not derived from the typical vascular bed to study the inflammatory response. Moreover, the confluency of the endothelium, which was maximal under the conditions used for our study, may also have been different. The fact that the microvascular human HDMEC cells exhibited vinculin localization along cell borders similar to endothelial cells of intact post-capillary venules indicates that the conditions used here were appropriate to study endothelial barrier regulation.

### RhoA is activated along intercellular junctions whereas no significant change in Rac1 activity or cAMP levels were detected

Thrombin can increase endothelial permeability by activating Ca^2+^ -/RhoA-signaling, which disrupts endothelial adherens junctions^47^. Similarly, several studies have shown the importance of RhoA signalling in histamine-induced leakage. Most recently, RhoA-deficient animals were protected against histamine-induced vascular permeability^26,47^. Our results support this notion (Fig. 8) since inhibition of ROCK effectively blocked histamine-induced barrier breakdown. However, how RhoA signaling induces the disruption of the endothelial barrier is less well defined. Because activation of RhoA GTPase is spatiotemporally controlled^26^ and overall activation as shown by GLISA may not accurately reflect activation at the site of cell junctions, we used Rho GTPase FRET sensors to visualize activation with high temporal and spatial resolution. These sensors change conformation and subsequent FRET efficiency depending on the local balance of guanine nucleotide exchange factors (GEFs) and GTPase-activating proteins (GAPs). Our data confirm that treatment with both thrombin or histamine induced activation of RhoA in the cytoplasm and also along cell borders. This is in line with the idea that RhoA has an important role in the regulation of endothelial barrier properties after treatment with inflammatory mediators.

In addition, it is well established that activated small GTPase Rac1 and the second messenger cAMP stabilize endothelial barrier properties^48^. For LPS and TNF-α, it has been shown that endothelial barrier breakdown temporally was closely associated with both decreased cAMP levels and Rac1 inactivation whereas RhoA activation was observed later^48,49^. Moreover, CNF-1 under conditions where it activated Rac1 and RhoA in parallel in cultured endothelium was effective to abrogate TNF-α-induced barrier breakdown in mesenteric post-capillary venules *in vivo* whereas Rho kinase inhibition was not. These observations suggest that inactivation of Rac1 rather than stimulation of the RhoA/Rho kinase pathway was the primary mechanism underlying TNF-α-mediated permeability increase.

Surprisingly, our results of the present study showed no differences in Rac1 activity or in cAMP concentrations after treatment with histamine. Therefore, we conclude that in contrast to the situation in sepsis, where LPS and TNF-α are relevant, alterations of cAMP levels as well as Rac1 activity are less important in anaphylaxis. Rather, acute inflammation appears to be primarily dependent on Ca^2+^ and activation of RhoA.

### Ca^2+^ signaling is required for RhoA activation, AJ reorganization and barrier dysfunction

Permeability mediators, including histamine, act on G_αq_-coupled GPCRs leading the activation of PLC-ß and the rapid mobilization of Ca^2+^, which promotes MLC phosphorylation and the stimulation of its contractile activity^27^. RhoA can be activated independently by H1R coupled to G_q/11_^28^. H1R also leads to an increase of intracellular Ca^2+^ concentration. As reported previousley^28^, we found that H1 receptor is required for histamine to induce permeability (Fig. 8). Our results revealed that both histamine and thrombin lead to a rapid Ca^2+^-influx accompanied by parallel reduction of TER. Chelation of Ca^2+^ by BAPTA-AM similar to inhibition of ROCK blocked the effects of histamine and thrombin on barrier function and AJ reorganization. Moreover, BAPTA-AM prevented activation of RhoA and similar to inhibition of ROCK blunted α18-catenin staining along junctions. These results indicate that Ca^2+^ -mediated RhoA activation is critical for endothelial barrier regulation (Fig. 8). The pivotal role of RhoA has been demonstrated previously^27^, however, in this study the authors concluded that RhoA was activated primarily independent of Ca^2+^. In addition, we observed that stress fiber formation in response to histamine was abrogated by chelation of Ca^2+^ but not by inhibition of Rho kinase. This supports the notion that in response to histamine stress fiber formation can be induced by Ca^2+^ in a manner independent of Rho kinase, most likely via MLCK^50^, and suggests that stress fibers are not directly involved in tension formation at AJ and endothelial barrier breakdown (Fig. 8).

## Conclusion

Therefore, we conclude that Ca^2+^ via RhoA/ROCK activation is critical for histamine-induced barrier disruption which correlated with tension-associated α18-catenin staining along endothelial AJ. These data suggests that the patterns of signaling mechanisms engaged in acute inflammatory barrier disruption differ to more delayed responses as seen in sepsis where reduction of endothelial cAMP and inactivation of Rac1 appears to be more relevant^5^. Furthermore, Rho-mediated tension may be involved in acute endothelial barrier disruption, at least *in vitro*.

## Methods

### Cell Culture and antibodies

All experiments were performed using Human Dermal Microvascular Endothelial Cells (HDMEC) (Promocell, Heidelberg, Germany). Cells were maintained in corresponding medium (Endothelial Cell Growth Medium MV; Promocell, Heidelberg, Germany) and used at passages between three and six. Vascular endothelial (VE-) cadherin was detected using goat-anti-VE-cadherin antibody purchased from Santa Cruz Biotechnology (Santa Cruz, USA). Vinculin was immunostained using mouse-pc-anti-vinculin antibody purchased from Abcam (Cambridge, UK). Rat α18-catenin antibody was contributed from Yonemura S. (RIKEN Center for Developmental Biology, Kobe,Jyogo, Japan)^30^. Filamentous actin (F-actin) was illuminated using an Alexa 488 phalloidin dye (Life Technologies, Karlsruhe, Germany). The addition of 4′,6′-diamidino-2-phenylindole (DAPI) was used to assess nuclear morphology. Corresponding secondary antibodies were purchased from Dianova (Hamburg, Germany) and were also used for control experiments (Supplementary Fig. S3).

### Test Reagents

Histamine and thrombin were purchased from Sigma-Aldrich (Munich, Germany). Histamine was used at 10 µM for the indicated times and thrombin was used at 10 U for 5 min. BAPTA-AM (Sigma-Aldrich, Germany) was preincubated for two hours at 5 µM. Diphenhydramine was used to inhibit activation of histamine receptor type I, and ranitidine was used for inhibition of histamine receptor type II. Both were preincubated at 1 µM and 10 µM, respectively, for 15 min. The ROCK inhibitor Y27632 (Sigma-Aldrich, Germany) was preincubated for 30 min at 10 µM.

### Transendothelial Electric Resistance Measurements

The barrier integrity of HDMECs was measured using an ECIS Z Theta system (Applied Biophysics, New York, U.S.A). HDMEC monolayers were grown to confluence on uncoated 8W10E electrodes. Immediately before the experiment, the medium was exchanged and the electrode was mounted on a holder inside an incubator at 37 °C with 5% CO_2_. Transendothelial resistance was measured at 4000 Hz every 10 seconds. Test reagents were diluted in pre-warmed medium. After recording 5 min of baseline values, the mediators were added.

### Immunostaining

HDMECs were grown to confluence on uncoated glass cover slips, treated with vehicle or test reagents and fixed with 4% formaldehyde in PBS for 10 min at room temperature. After washing and permeabilization using 0.1% Triton X-100 in PBS, the cells were blocked with 10% normal goat or donkey serum and 1% BSA in PBS. The respective primary antibody was incubated overnight at 1:100 in PBS at 4 °C. After washing, cells were treated with respective secondary antibodies coupled to Cy2, Cy3 or Cy5 for one hour and then embedded in n-propyl gallate. F-actin was stained using an Alexa 488 phalloidin dye added to the diluted secondary antibody at 1:100. Using the fixation protocol described above, patterns of actin filaments under control conditions resembled those observed in previous studies^49,51,52^. However, we cannot guarantee that all peripheral filaments were preserved when compared to an unfixed state, which we believe is not important for this study.

Stained monolayers were sequentially illuminated using a Leica SP5 confocal microscope (Mannheim, Germany) with an HCY PL APO Lambda blue 63 × 1.4 oil immersion objective and a zoom of 2x. Cy3 was illuminated with a wavelength of 542 nm and Cy5 with 633 nm. The same microscope settings were used under all conditions. Secondary antibody controls were performed for all co-immunostainings (Supplementary Fig. S3).

To calculate the Pearson’s correlation coefficient confocal images were analysed using Image Processing and Analysis in Java´s (Image J) segmented line and plot profile tools. A line (110 pixels length, 2 pixels width) was drawn along cell borders based either on F-actin or vinculin distribution. Subsequently, plot profiles were generated using the built-in tool. Pixel gray values for both proteins of interest obtained from these plots were transferred to Excel and used for calculations of the Pearson’s correlation coefficient.

### G-Protein ELISA (GLISA)/cAMP-ELISA

Intracellular concentrations of GTP-bound Rac1 and RhoA proteins were determined using commercially available ELISA kits purchased from Cytoskeleton (Denver, U.S.A). The assay was performed according to the manufacturer’s instructions. The technique was used as described previously^33^, cAMP concentrations were measured by cAMP enzyme linked immunosorbent assay (CA-200) (Sigma-Aldrich, St. Louis, U.S.A.) as described previously^53^.

### Fluorescence Resonance Energy Transfer (FRET)

Spatiotemporal activation of RhoA was determined using a RhoA-Ras and interacting protein chimeric unit (Raichu) –probe (a kind gift of Miyazaki J., Kyoto University, Japan)^54,55^ consisisting of four modules: a donor (CFP), an acceptor (YFP), a Rho binding domain (RBD) and the Rho GTPase itself. Upon exchange of GDP to GTP, the RBD binds the GTP-RhoA complex and the distance between the donor and acceptor decreases, increasing the energy transfer from the donor to an acceptor when illuminated at donor excitation wavelength. The RhoA-Raichu probe is contained in a transfectable plasmid. The plasmid was transfected with jetPRIME (VWR) into HDMECs at 80% confluence, grown to a confluent monolayer and then subjected to FRET-measurements. After the addition of mediators, the cells were fixed using 4% PFA in PBS for 20 min. Using standard acceptor photobleaching protocol^56^, YFP was bleached for 3 min at 514 nm and CFP intensity was recorded before and after bleaching. Processing was performed in ImageJ and absolute FRET efficiency values were recorded.

### FURA-2 AM Measurements

Intracellular Ca^2+^ concentrations were detected by radiometric analysis of FURA-2-AM (Thermo-Fisher, Massachusetts, USA). Cells were incubated in FURA-containing medium for 45 min, and then the medium was exchanged. After a 15 min rest period to allow cleavage of the ester, the cells were illuminated every 3 seconds and the signal was detected at 340 nm/380 nm. After 20 seconds of baseline detection, reagents were added.

### Microperfusion of rat venules

We perfused single postcapillary venules in the rat mesentery using the modified Landis technique^3,57,58^. Wistar rats (provided from Janvier Labs, Saint-Berhevin Cedex) with body weights between 280 g and 350 g were anesthetized by subcutaneous injection of ketamine (60.0–75.0 mg/kg) and medetomidine (0.25–0.5 mg/kg). Then small unbranched microvessels with convergent flow were cannulated using sharp glass micropipettes and perfused continuously with mammalian ringer solution containing 10 mg/ml bovine serum albumin (BSA) with or without 10 µm/l histamine dihydrochloride. Further addition of diluted erythrocyte concentrate allowed visualization of correct placement and flow. After 5 min of perfusion, the rats were euthanized and the intact mesentery was washed and fixed immediately in 4% formaldehyde in PBS. The mesentery was then spread on adhesive glass slides, permeabilized with 0.1% Triton in PBS and blocked using BSA/NDS. Further immunostaining for VE-cadherin, vinculin and F-actin was performed as described above. Illumination was performed by taking z-stacks with a confocal microscope (Leica SP5 confocal microscope) using a 63 × 1.4 water immersion objective. All rats were kept under conditions that conformed to the regulations of the Regierung von Oberbayern and all experiments were performed in accordance with relevant guidelines and regulations. The experimental protocol was approved by the Regierung von Oberbayern.

### Statistics

Immunofluorescence experiments were quantified using CellProfiler (CarpenterLab, Cambridge, USA). After background subtraction, cell borders were segmented and the mean fluorescence intensity recorded^59^. For automated measurements of fluorescence intensity in the cytosol, the area of the segmented cell borders was subtracted from each image and mean intensity was calculated. The data analysis was carried out using R^60^ (R Foundation, Vienna, Austria) and OriginPro 2017 g (OriginLab Corporation, Northampton, USA). Statistical analysis was carried out using one-way ANOVA followed by Holm–Bonferroni corrections for multiple comparisons. All data are presented as the mean ± standard error of the mean. The results were considered statistically significant at p < 0.05.

## Electronic supplementary material

Supplementary Figures
