# Supplementary material for: Histamine causes endothelial barrier disruption via Ca2+-mediated RhoA activation and tension at adherens junctions
Source: Sci Rep. 2018 Sep 5;8:13229. doi: 10.1038/s41598-018-31408-3 (PMC6125323; doi:10.1038/s41598-018-31408-3)
Supplement: Supplementary file 1 — Supplementary Figures [file 41598_2018_31408_MOESM1_ESM.pdf]

Histamine causes endothelial barrier disruption via Ca^{2+}-mediated RhoA activation and tension at adherens junctions

Kugelmann Daniela[1], Rotkopf Lukas Thomas[1], Radeva Mariya Yosifova[1], Garcia-Ponce Alexander[1], Walter Elias[1], Waschke Jens[1*]

[1]Institute of Anatomy and Cell Biology Department I, Ludwig-Maximilians-Universität München, Munich, Germany.

[*]Corresponding author: e-mail: jens.waschke@med.uni-muenchen.de

Supplementary Figure S1

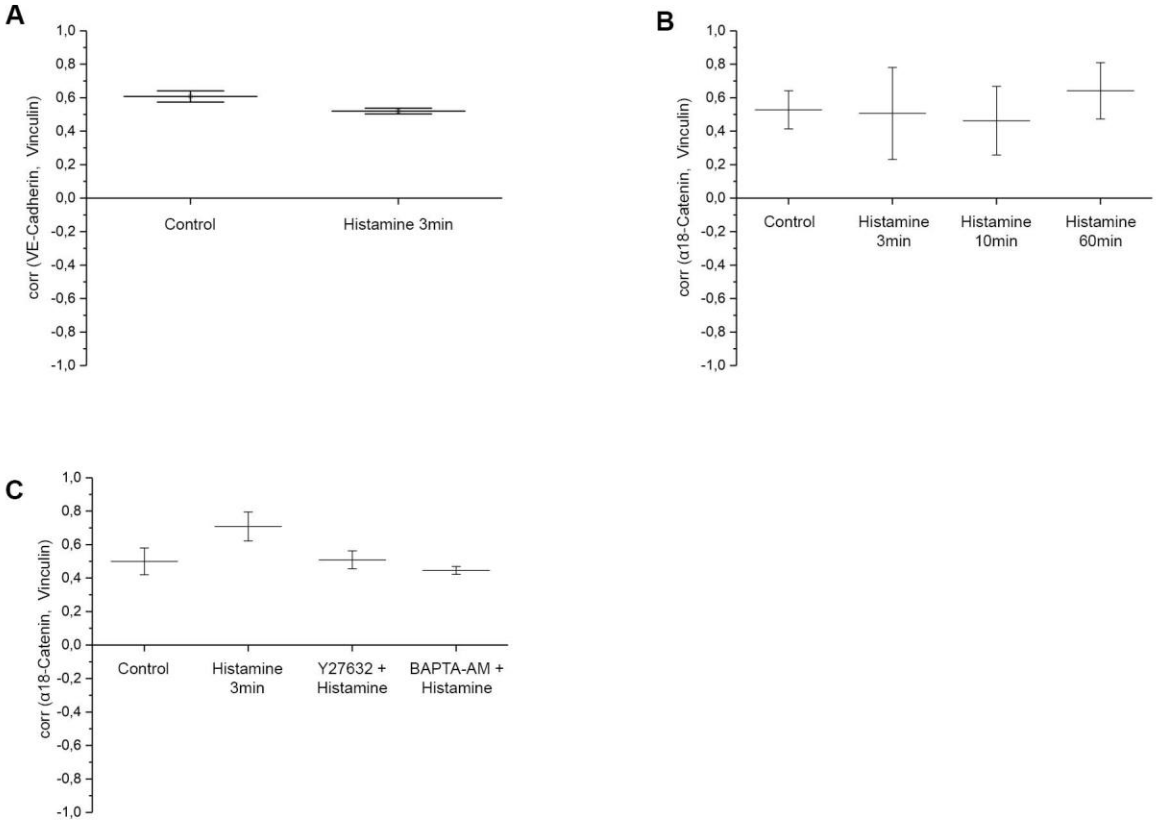

Co-localizations in immunostainings evaluated by Pearson's correlation coefficient.
Pearsons correlation coefficients were calculated on immunostainings shown in the main figures (Fig.1 D+E, Fig. 2 A+B, Fig. 6 A+B). Positive values until +1 demonstrate a positive correlation between two different stainings indicating protein co-localizations. Neither the treatment with histamine nor the treatment with histamine in conjunction with inhibitory mediators (Y27632 and BAPTA-AM) interfered with co-localizations of VE-cadherin (A) or α18-catenin (B, C) to vinculin.

Supplementary Figure S2

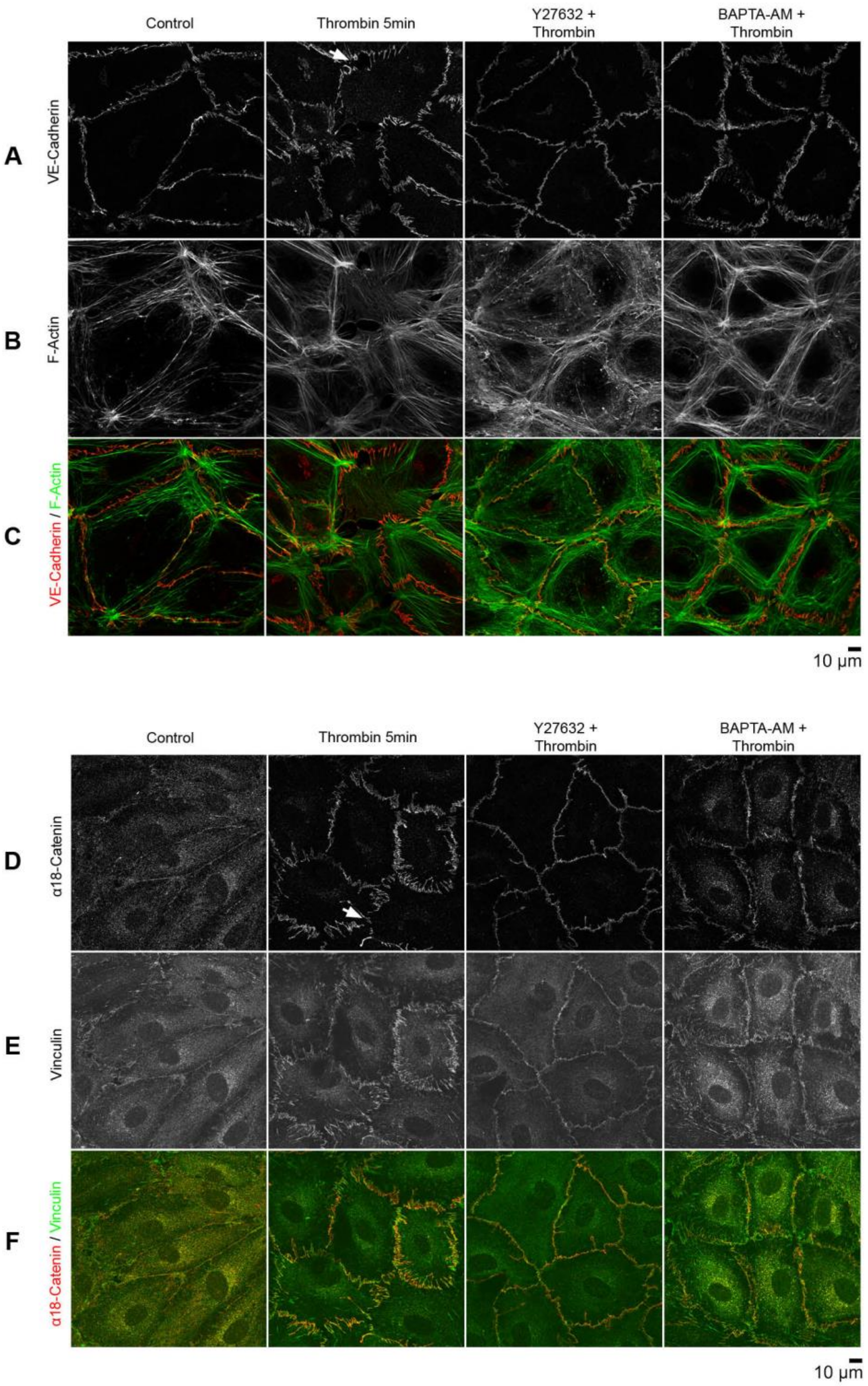

Thrombin induced barrier dysfunction was reduced by inhibition of ROCK and Ca^{2+} signaling. HDMEC monolayers treated with thrombin (5 min) in the absence or presence of inhibitory mediators (Y27632 and BAPTA-AM) were co-immunostained with either (A-C) VE-cadherin and F-actin or (D-F) α18-catenin and vinculin (n=3 independent experiments). Similarly to histamine, thrombin induced formation of gaps (A+D, arrows) and stress fibers (B). The effects were abolished by application of Y27632 and BAPTA-AM. (D) A stronger α18-catenin staining along the membrane, was induced by thrombin. However, the inhibitors were able to reduce the thrombin effect. (E) Vinculin staining was detectable on the membrane under all conditions.

Supplementary Figure S3

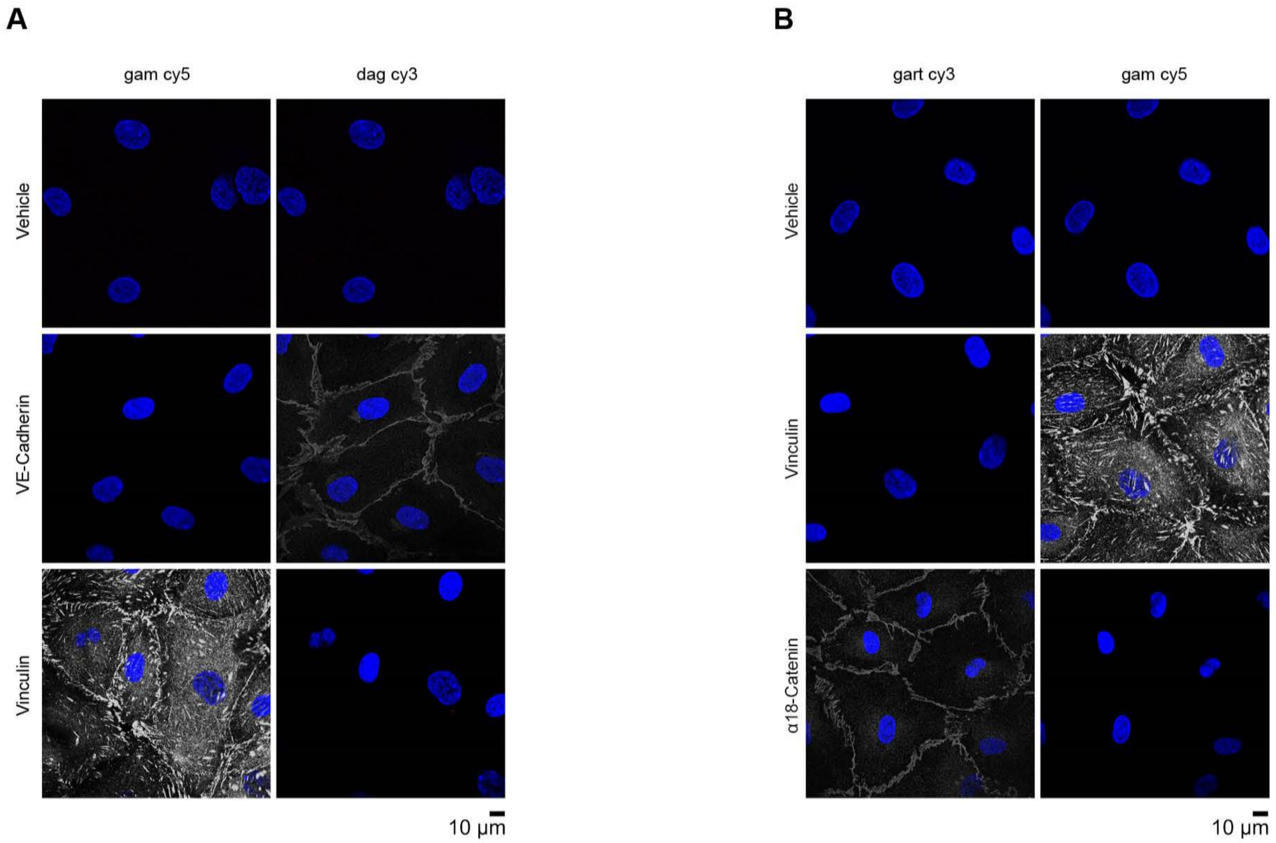

Secondary antibody controls for co-immunostainings

(A, B) All conditions represent incubations with different combination of secondary antibodies. In contrast to the vehicles in all other conditions the cells were pretreated with primary antibodies as indicated. No cross reaction was observed with the secondary antibodies not corresponding to the respective primary antibody. Nuclei were counterstained by 4′.6-Diamidin-2-phenylindol (DAPI). Gam = goat anti-mouse, dag = donkey anti-goat, gart = goat anti-rat.
